# Supplementary material for: A new test to detect impairments of sequential visuospatial memory due to lesions of the temporal lobe
Source: PLoS One. 2022 Jul 29;17(7):e0272365. doi: 10.1371/journal.pone.0272365 (PMC9337684; doi:10.1371/journal.pone.0272365)
Supplement: S1 Table — (PDF) [file pone.0272365.s001.pdf]

S1 Table. Individual performances in DILSS, Corsi block-task, and neuropsychological examination. *Diagnosis*: (0: control; 1: patient); *ID*: subject identifier; *p_i*: initial recall probability; *p_f*: final recall probability; *speed*: initial learning speed; *Corsi block-span*: average of the forward and backward Corsi block-span; *CVLT short delay*: number of recalled words after 5 presentations; *CVLT long delay*: number of recalled words after 20 min; *ROCFT*: immediate recall score of the Rey-Osterrieth Complex Figure Test; Handedness: Oldfield test (<0: left handed; >0: right handed); MoCA: score of the Montreal Cognitive Assessment test; BDI: score of the Beck Depression Inventory test. Session 2 and Session 3 were performed 30 min and one week after Session 1

Diagnosis	*ID*	Session 1			Session 2		Session 3		*Corsi block span*	*CVLT short delay*	*CVLT long delay*	*ROCFT immediate recall*	*Handedness (Oldfield)*	*MoCA*	*BDI*
		p_i (%)	*p_f* (%)	*speed* (targets/trial)	*p_i* (%)	*speed* (targets/trial)	*p_i* (%)	*speed* (targets/trial)							
0	1	15.46	99.99	1.37	96.15	0.14	55.89	1.17	6.0	-	-	-	100	28	0
0	2	9.17	100.00	2.74	100.00	0.00	100.00	0.00	5.5	-	-	-	100	-	4
0	3	15.39	95.12	0.67	90.11	0.12	83.04	0.30	5.5	-	-	-	60	29	2
0	7	13.69	99.85	1.06	94.49	0.03	67.24	0.78	6.5	-	-	-	-100	30	2
0	10	74.24	99.97	0.60	100.00	0.00	100.00	0.00	7.5	-	-	-	100	30	2
0	11	21.90	58.25	0.26	57.72	1.12	48.24	0.54	7.0	-	-	-	89	28	3
0	13	11.09	100.00	1.69	98.55	0.04	85.00	0.60	7.0	-	-	-	-100	30	1
0	15	41.32	100.00	1.84	100.00	0.00	80.00	0.80	5.5	-	-	-	79	30	0
0	16	20.62	100.00	2.46	80.00	0.80	90.00	0.40	7.5	-	-	-	79	29	12
0	17	13.92	100.00	1.77	100.00	0.00	-	-	6.0	-	-	-	90	30	2
0	19	3.54	100.00	1.86	100.00	0.00	99.46	0.00	7.5	-	-	-	90	27	10
0	21	35.56	100.00	2.19	100.00	0.00	98.26	0.02	7.5	-	-	-	90	29	3
0	23	14.34	100.00	1.69	100.00	0.00	96.42	0.06	6.0	-	-	-	100	30	4
0	26	21.31	95.72	0.72	95.00	0.20	100.00	0.00	6.5	-	-	-	80	27	0
1	5	14.88	99.18	0.90	94.39	0.20	14.61	2.02	6.0	13	14	19.5	100	29	0
1	6	14.82	98.52	0.82	100.00	0.00	46.26	0.76	6.5	11	9	-	90	27	1
1	9	17.09	75.26	0.40	76.05	0.44	26.88	1.43	6.5	12	15	24.0	70	29	12
1	12	41.86	44.54	0.02	35.19	0.18	-	-	5.0	15	16	20.5	100	28	11
1	14	23.94	38.14	0.11	35.37	0.21	33.43	0.16	5.5	8	7	13.0	100	28	4
1	18	19.40	49.04	0.21	36.97	1.33	69.89	0.29	5.5	14	16	20.0	100	27	7
1	20	11.19	59.44	0.27	55.10	0.65	-	-	4.5	13	14	11.5	100	30	2
1	22	16.49	68.21	0.34	54.01	0.40	21.35	0.72	5.5	-	-	-	100	27	1
1	24	34.77	84.41	0.45	90.13	0.04	65.50	0.57	5.5	12	13	21.0	100	27	5
1	27	21.14	41.81	0.15	39.29	0.00	30.82	0.32	5.5	14	12	20.0	100	29	0
1	28	5.51	97.56	0.57	90.00	0.40	67.63	0.94	5.5	12	13	20.0	0	27	1

1	29	24.12	89.55	0.57	60.68	1.36	77.67	0.44	7.5	14	14	26.0	100	30	6
1	30	15.35	90.31	0.57	85.46	0.38	72.59	0.84	6.0	-	-	-	80	27	5
