# Supplementary material for: A new test to detect impairments of sequential visuospatial memory due to lesions of the temporal lobe
Source: PLoS One. 2022 Jul 29;17(7):e0272365. doi: 10.1371/journal.pone.0272365 (PMC9337684; doi:10.1371/journal.pone.0272365)
Supplement: Attachment — Submitted filename: ResponseLetter.docx [file pone.0272365.s002.docx]

We would like to thank the editor and the reviewers for their comments on our manuscript. In the following we respond to the reviewers point by point and describe the changes we made in the revised manuscript. The reviewers’ comments are printed in blue and our responses in black. Page numbers and line numbers refer to the version of the revised manuscript with all changes with respect to the original version marked in red.

**Editor Comments:**

I echo the reviewers' comments. Particularly, the distinct group-difference in age and possible cognitive decline at the presurgical stage should be appropriately addressed, although the topic of visual episodic memory in TLE is interesting.

We addressed these points in our answers to the points 1 and 3 of reviewer #2 and in the new section “Limitations” in the Discussion.

**Review Comments to the Author**

Reviewer #1: The authors evaluated visuospatial episodic memory of patients with epilepsy after temporal lobo resection and healthy controls using sequential visuospatial memory test. This new neuropsychological test showed impaired visuospatial memory in patients after right side resection in the first evaluation, but retention rate at one week later was worse in patients after left side resection. The standard neuropsychological tests failed to show the difference. The authors focused on visuospatial memory function which is sometimes undervalued in the perioperative workup and successfully demonstrated the left-right difference of the hippocampal function using a novel sequential test developed to specifically extract episodic memory. This study is valuable and is worth publishing in PLO SONE, if the following concerns are appropriately addressed.

Thank you for this positive evaluation.

Methods

L130.	Please clarify the inclusion criteria of the patients after epilepsy surgery. Did you include all patients who underwent epilepsy surgery, became seizure free, and agreed to join the study?

The details of the recruitment and inclusion criteria were added (L132-135).

L133.	Typo? The information on seizure onset is provided in S1, not S2. Anyway, the demographic data shown in S1 is important to interpret the results. S1 should be in the main text, not in the supplementary.

We apologize for the typo and have corrected it (L 137). Following the suggestion of the reviewer, we moved Table S1 from the supporting information to the main manuscript.

L135.	Was the language dominance determined solely by handedness? If so, uncertainty in the language dominance should be mentioned as an unignorable limitation of this study.

We added the statement that “language dominance was not determined separately” and added a reference to the study of Knecht et al. (2000) showing that 96% of healthy right-handers have left-hemispheric speech dominance (L139-L141).

L201.	The phrase "training sessions" implies that there will be a test session. This is just a comment.

We removed the word “training” to avoid misunderstandings (L206). In fact, Session 1 is a training session and the two subsequent sessions serve as tests.

Discussion

L497.	The authors wrote, "due to TLE resection". But, it would be inappropriate to attribute the memory impairment solely to resection, as it would had originally been impaired due to prolonged epilepsy.

The attribute “due to TLE resection” was removed (L501-502)

S1.	According to the table, patient 6 and 9 underwent tumor resection. Were their hippocampi resected? Please clarify whether the hippocampus of each patient was removed or not.

The information that “the hippocampus was resected in all patients” was added in the text (L142) and in the figure legend of Fig. 1.

Fig1.	It is unclear whether the hippocampus of each patient was removed from the slices in this figure. For example, the hippocampus of patient 6 appears to remain. Mostly, the slices seem too close to the middle base. The petrosal bone should not be in the slice in order to show the hippocampus correctly.

We added the sentence “To illustrate the size of the resections, the slices are centered on their largest extent, not on the hippocampus” to the legend of Fig. 1.

Reviewer #2: Thomas Eggert al. applied the DILSS-task, a new method to detect impairments of sequential visuospatial memory in 13 patients with temporal lobe epilepsy and 14 healthy controls. The result indicated that learning speed was reduced in patients, also right-sided lesions induced worse impairments than left-sided lesions, which show the right hemispheric dominance in visuospatial processing.

Limitations:

1) Treatments, age would influence the results and hence should be controlled. In line 130, the average age of the control group was 29.5 ± 9.7, and that of the patient group was 40.2 ± 11.1. The age factor was not controlled; In addition, the treatment plan and effect before and after right temporal lobectomy and left temporal lobectomy were not specified in detail. This factor may have an impact on the evaluation of patients' memory function.

We agree with the reviewer that the lack of controlled factors limits our study in several ways, which are now described in the new "Limitations" section added to the Discussion (L586-601). At this point, we clarify that our data are not sufficient to validate DILSS as a test for clinical use and that this was not the purpose of the current study. Our aim was to investigate whether a newly developed memory test was more sensitive in this patient group compared to other neuropsychological tests. Leaving factors like the exact medication of the patients uncontrolled probably increased the inter-subject variability of the patient group and therefore made it more difficult to detect differences with respect to the controls. Thus, even though our study is not able to determine the role of such factors in detail, it shows nonetheless that our test reached a high sensitivity to distinguish this patient group from controls.

The new section also discusses the question of age effects raised by the reviewer. This is a more critical point, because the mean age of our patient group was higher than that of the controls. Memory performance generally deteriorates with age as has been reported by many studies for verbal memory [1, 2] and also for the Rey-Osterieth Complex Figure Test (ROCFT) [3]. These studies generally show a significant decline in performance above the age of 50 but relatively stable performance between ages 20 and 40. For example, the study of Gallagher and Burke (3) showed that the older age groups (50-69 yrs) performed significantly worse in the recall than the younger age groups (20-39 yrs). However, in the age range between 20 and 49 yrs, age effects did not reach significance (F(2,57)=1.56; p= 0.22, see Figure R1). Based on these results from the ROCFT, an age difference between two groups such as our patients (age 40.2±11.1) and controls (29.5±9.7 yrs) does not explain the highly significant difference in learning speed we observed. Consequently, this age difference does not compromise the main conclusion of the study.

**Figure R1:** Age dependence of a large group (N=115) of healthy subjects in the recall performance of ROCFT (replotted from Gallagher and Burke (3)). The bars and whiskers show mean and standard deviation of the raw score in the recall, performed immediately after completion of the copy. The age effect across all age groups was highly significant (F(5,105)=7.71, p<0.001), due to impaired performance for age>50. Across the three age groups between 20 and 50 yrs, the age effect on the recall score did not reach significance (F(2,57)=1.56; p=0.22).

2）There is no indication whether the test was conducted by the same observer, and there may be observer bias.

The evaluation of the recorded pointing movements was analyzed by a computer and completely automated as described in the Methods. The instructions were standardized and given by same examiner (L170-171).

3）There were only 13 patients and 14 control in the groups, the sample size was too small to support the result.

The reviewer expresses here that descriptive parameters such as averages obtained from small samples do not allow conclusions about averages of larger populations. This doubt is certainly justified, since population averages and group differences can be determined with higher precision in large samples. However, it should also be noted that for clinical diagnosis, the group difference of a parameter is often less interesting than the discrimination power that it can achieve. Using ROC-analysis, we demonstrated in a relatively small group that the learning speed in DILSS was quite efficient to discriminate our patients type from healthy controls. This doesn't mean, of course, that the discriminative power of this parameter will transfer unchanged to larger samples. The significant group effects of our study provide a motivation to investigate our learning test in a larger population in the future. We fully agree with the reviewer that our study does not reach the level of generalizability expected of a clinical trial, and we have never claimed that it did. We hope that the new section in the discussion (L586-601) helps to clarify this point.

Other comments:

"CVL" should be changed to "CVLT" in line 513.

Done. (L518)

# References

1.	Baltes PB, Lindenberger U. Emergence of a powerful connection between sensory and cognitive functions across the adult life span: a new window to the study of cognitive aging? Psychology and aging. 1997;12(1):12.

2.	Kramer JH, Yaffe K, Lengenfelder J, Delis DC. Age and gender interactions on verbal memory performance. Journal of the International Neuropsychological Society. 2003;9(1):97-102. doi: 10.1017/s1355617703910113. PubMed PMID: 12570363.

3.	Gallagher C, Burke T. Age, gender and IQ effects on the Rey-Osterrieth Complex Figure Test. Br J Clin Psychol. 2007;46(Pt 1):35-45. doi: 10.1348/014466506x106047. PubMed PMID: 17472200.
